# Monthly Periscope

**Published:** 1853-10

**Authors:** 


### EDITORIAL DEPARTMENT.

*Monthly Periscope.— Medical Colleges.*—The most prominent engines of medical progress are the colleges. It has, of late, grown somewhat the fashion to decry them, and our whole system of medical education. We doubt the correctness of these complaints. Were the individual members of the profession as true to its interests as are the schools, we should be well satisfied. As our government institutions are now organized, and in the present tendencies of the popular mind, we are disposed to consider our present educational system, the one best adapted to the situation, and the wants of the country. This system has, for its characteristic points:

First—a complete independence from government control, or patronage.

Second—an entire dependence on the respect and good-will of the profession for support.

Third—an active competition of the schools with each other.

We regard all these as incentives to excellence, which cannot be reached under any other system attainable in this country. The principle of independence from government control involves many important considerations. The success of medical teaching in Paris, Berlin, and Vienna, has been often quoted as an argument for the establishment of a similar system here. But so essential is the difference in our political institutions, that an examination of the subject will show its imperfect adaptation to our wants; and illustrate the fact, that those very schools so justly celebrated, would not flourish if transplanted to this country.

The first requirement for the success of a government school, is its location in a large capital. There are gathered facilities for clinical instruction, for dissections, and there are those inducements of large fields for private practice, which alone can call together the first order of talent. The immense size, and peculiar social organization of Paris, are the principal causes of the excellence of its schools. To show that the government system is insufficient and unsuccessful in Europe, we shall here interpolate a few extracts from the published letters of a distinguished medical observer and editor, written from the various cities and countries which he mentions. Speaking of the University School of Brussels, he says:

"A poorer cabinet of anatomy, morbid specimens, and wax models, has not come under recognition in any country."

From Antwerp: "Not a syllable has yet been advanced in reference to hospitals, medicine, or its interests in Antwerp, for the reason that there is little to record."

From Leyden: "Instead of the thousands of students who once crowded the town, the number now averages about seventy."

From Amsterdam: "With a good collection, an able board of professors, and the *sustaining power of government*, its system of influence is small."

Writing from Baden-Baden in reference to German schools as a class, he says:

"Circumstances, in the profession of medicine, make the man even in Europe. A very few rise to distinction by their energy of character, but the first positions are in the safe keeping of family influence. When a biographer speaks of this, or that one, being called to a professorship, it would be far more honest to declare that through the controlling activity of friends, or relatives, he was raised over the heads of competitors, whose only qualification was merit, a kind of coin that is quite as much below par in Germany, as in other countries. In France — Paris especially — there is a splendid exception to the monopolizing spirit."

And so on throughout the various cities of Europe, and even in Asia, at Constantinople, and in Africa, at Cairo, our author finds occasion, evidently to his own surprise, to condemn the dependence of medical schools upon government support.

It is, then, proposed to adopt in this country, a system which has already proved a failure in Europe. Would it succeed better if transplanted to a republican soil? We have already sufficiently shown that the plan can only flourish in great capitals. In New York, in New Orleans, and possibly in Philadelphia, this condition could be complied with. But, if family influence is so ruinous in Europe, would it be less so here? The government plan requires such permanency in the appointing power as can only be had in monarchical countries. The professorial appointments should be free from *political* changes, and their influence. In a word, as our politics are at present carried on by a system of partizan rewards and punishments, no state schools will remain long free from this deteriorating influence; and with the constant changes in governors and cabinets, an equally constant change in teachers will obtain, and active political character be looked upon as the first requisite in a medical teacher. This is not a theoretical, but a practical and imminent danger; and the combination of family with political influence, would result in the elevation of the sons of great men, and small practitioners, to the professorial chairs.

The system of *concours* is one necessary to the well-being of government schools which will not be adopted in this country. We say will not, because it would clash with political intrigue, and party spoils. But were it adopted it is still subject to family and personal influence. It is the only, but the feeble guaranty against outside collusion. Without it such collusion is inevitable — with it, it is probable.

A large salary is another condition of success. Talent has a cash value, and the salaries should be such as to fill the chairs with an array of superior men. A man of real eminence, of active energy, and true progress, will not for a thousand, or two thousand dollars yearly, consign himself to what must soon become the obscure and forgotten position of professor in a sleepy government school.

Another thing may be mentioned: The opening of free schools of medicine would certainly have little tendency to improve the character of the profession. Medicine would become the refuge of broken down clergymen, and disgraced lawyers; of all those whose dislike to honest manual labor would lead them to avoid it. In a country like this, every high-spirited and honest young man should be able to enter the portals of the profession by the fruits of his own labor. The discipline of the struggle for success, and the manly feeling of pride which should reward that success, must be far preferable to the charity-boy feeling, inseparable from benefits unpaid for.

We have thus enumerated the advantages attendant on our present system, and the disadvantages, viz: want of proper location and clinical teaching, the danger of political and family influence, the absence of the "*concours*" method of appointment, and of permanence and stability in the appointing power; the smallness of the salaries likely to be paid, and the consequent inferiority of the teaching; and finally, the loss of influence and character to the profession by the admission to it of unworthy members, attracted by the cheapness of the diploma. Until these objections are overcome, and this requirement complied with, no state school should hold a position of equality with those worth paying for — better taught, and better supplied with the collateral means of instruction.

We have mentioned the dependence on the good-will and respect of the profession, as a good feature of our schools as now organized. It has obvious advantages. Schools are subjected to the rigid censorship of public opinion, which holds in its hands the most effectual weapon of punishment for any malfeasance — a withdrawal of patronage. But here, as in Germany and elsewhere, a state school in favor with the authorities, could hold out against any force of professional sentiment.

Finally, we have to speak of free competition as a necessity of the age, and the country. The whole current of our legislation, and the true spirit of our institutions is against monopolies, and the increase of governmental appointments. Government builds no more railroads, it digs no more canals, and it endows no more colleges. And it is to free competition, that we are indebted for our present standard of excellence. A competition for large classes, and the money to be got from them, is the surest and best stimulus to high perfection. Without this stimulus, with a salary independent of the popularity of the school, and without competitional incentives to labor, a state of hebetude and "old fogyism" is inevitable. One of two things must happen. Give to a government school freedom from political influence, a large pecuniary support, and permanency in its professorial chairs, and its teachers will become indolent and stationary. Or, subject them to partizan influence, and frequent changes in the chairs, and their desks will be filled by men of merit as stump speakers, and political toadies, without regard to professional attainment or skill. The condition of the Canadian government schools is an instance of the first horn of the dilemma. The establishment of state schools in this country will furnish the second.

*Statistics of Cerebral Disease.*—We abstract and condense from the Dublin Medical Press, the following tabular view of the phenomena of fatal cases (which only are mentioned) of brain diseases, by A. W. Barclay, M. D.:

*Of scrofulous inflammation* there were 28 cases.

| | |
|---|---|
| Under 15 years of age, . . | 9 cases. |
| From 15 years to 20, . . . | 5 " |
| " 20 " 25, . . | 7 " |
| " 25 " 30, . . . | 4 " |
| " 30 " 40, . . | 2 " |
| " 40 " 50, . . . | 1 " |
| Over 50 " . . . | 1 " |

It will be noticed that this arrangement foots 29 cases, instead of 28. Another fact will at once attract attention, viz., the number of cases occurring in adult life. We had regarded scrofulous inflammation, or to use a more definite term, tubercular meningitis, as peculiar to infantile life, and have been in the habit of assuring parents, anxious concerning big headed and strumous children, that the period of greatest danger terminated with seven years of age. As usual, statistics differ from opinions less carefully formed. In regard to the symptoms present in these cases, we find the following:

They were "sometimes slowly and insidiously developed, at other times setting in with unexpected violence; while no point in the history of the cases could be learned in any way accounting for their origin." * * *

"In 11 cases pain in the head was the earliest symptom; in 6 delirium was the first prominent symptom; in 2 convulsions commenced as the first evidence of mischief. In 11 out of 28 cases, *pain in the head was not complained of;* delirium was absent in 8 cases of this class. Convulsions may be said to be the rule, and their absence the exception, in childhood. Among the 28 cases there was no exception to this under the age of 13."

The absence of pain in the head we have italicized. It accords with our own recollections, and is a most important fact in diagnosis. The rule as to convulsions in childhood, is also very interesting in a physiological point of view. We have only room for the following as to *simple inflammation and congestion.* The striking difference in the age of the patients is particularly valuable, as all these cases are based upon *post-mortem* appearances:

"The earliest period of inflammation was 16 years, and only 3 were reported under the 27th year; the period at which all but 5 of the scrofulous cases had terminated. Classed in periods of 10 years there were

| | | | | |
|---|---|---|---|---|
| From 15 years | to 25 years, | 3 cases. | | |
| " 25 | " 35 | " | 8 | " |
| " 35 | " 45 | " | 4 | " |
| Over 45 | " there were | | 3 | " |

The oldest occurring at 57. Of the 18 cases only 4 were females."

*Yellow Fever in New Orleans.*—The N. O. Medical and Surgical Journal gives a tabular statement of the mortality of the recent epidemic in that city. Our space does not permit the insertion of the entire article. We therefore condense the table, and subjoin below a word of apology from the editor, suggested by the small amount of original matter in that number. Our readers will notice in that simple statement, a pathos which will awake their sympathies. It tells a tale of such labor, anxiety, and peril, as only the physician knows.

The fatality of the present epidemic is attributed to the great number (estimated at 30,000,) of unacclimated persons who had accumulated in the city since the last epidemic in 1847. Its type was nearly the same as that of previous years. As usual, many cases had but little febrile movement, anterior to the appearance of black vomit, and consequently no treatment was instituted until the case was hopeless. In other cases, the invasion was so sudden, and the system so saturated with the morbific cause, that all remedies were of no avail. Four physicians perished by the epidemic: Dr. A. R. Nye, Dr. Jacobson, Dr. A. C. Robertson, and Dr. Friend.

| DEATHS. | YELLOW FEVER. | OTHER DISEASES. | NOT STATED. |
|---|---|---|---|
| During the week ending May 28th, . . . | 1 | 139 | |
| " 4 weeks " June 25th, . . . . | 21 | 604 | |
| " 5 " " July 31st, . . . | 1427 | 741 | 42 |
| " the week ending August 7th, . . . | 967 | 150 | 69 |
| " " " " " 14th, . . . | 1288 | 163 | 75 |
| " " " " " 21st, . . . | 1346 | 154 | 75 |
| " " " " " 26th, . . . | 884 | 124 | 66 |
| Total, . . . . | 5934 | 2075 | 327 |

The whole number of deaths from all causes, from May 21 to August 26, was 8336. The greatest number of deaths from the fever in any one day, occurred on Aug. 21; on this day the mortality from this cause was 239.

" *Ourselves.*—Our readers must bear with us for any short comings in this number of our Journal; our attention has been given for the most part, to those stricken down by the epidemic during the last six weeks; and we have had neither the time, nor that calmness of spirit which would enable us to collect and arrange our thoughts for publication. Our energies, both of body and mind, have been taxed to the utmost of their power and endurance, in combating this terrible epidemic; often for hours and hours has sleep, so necessary to our well-being, been denied us; and when a few moments of leisure were vouchsafed unto us, the mind was too weary, and the heart too sad to perform our editorial duties. Need we say more by way of apology on this subject; nevertheless, we think this number possesses a good deal of interesting matter. We shall do better next time."—*Ed. N. O. Med. and Surg. Journal.*

*Galactagogue and Emmenagogue effects of warm and stimulating applications to the Mammæ.*—In the American Journal of Medical Sciences, is an article by John Roso Cormack, M. D., on the above subject. He details cases wherein stimulating liniments upon the breasts excited the flow of milk, and also other cases in which sinapisms to the breasts acted powerfully as emmenagogues. With this latter application we are familiar. In our own hands, it has thus far acted with a certainty which has not attended the usual emmenagogues. In acute suppression it has thus far never failed. In one case, after an amenorrhœa of eight months standing, it was also successful. A large sinapism was placed upon one mamma, and a mustard pediluvium was used. The sinapism remained on the breast as long as it was tolerable, and excited much deep-seated inflammatory action, and swelling of the gland. With the subsidence of this swelling, (and this is the usual course,) the menses reappeared. Sometimes a week elapses, but oftener only two or three days, before the discharge is reinstated. In another case where the function had been absent for eighteen months, and the system much—and at last fatally—broken down by bronchial disease, we made the attempt to restore the function by this means. She took for a fortnight the iodide of iron. No menstrual nisus being perceptible at the end of this time, we blistered the inside of the thigh, applied a large sinapism to the breast, and used as auxiliaries the mustard footbath, and flannel clothing about the hips. By these means much lumbar and uterine pain was excited, and it seemed probable that we were to succeed. The patient herself was sure, from the amount and character of pain, that the secretion was about to be renewed; but after a week all these symptoms passed away without result. She died suddenly a month afterward. The case, though unsuccessful, illustrates the power of the remedy in a striking manner.

*Dr. Cartwright, Mrs. Willard, Western Editors, and the "battle of the evidences."*—We have above a most comprehensive subject for an article. Mrs. Willard has propounded a new theory of the circulation, which she or her friends designate as the "Willardian" theory. Dr. Cartwright espouses the new doctrine; and by reference to his lexicon, he shares in the discovery, by inventing it a name, and the whole is christened by Dr. C., (after due research in the aforesaid lexicon,) "the Willardian discovery of the hæmatokinetic, or new motive power of the blood." Whether the blood has recently acquired, as Dr. C.'s phraseology would indicate, any new motive power, we are unable to say—indeed it is for the purpose of condensing our own opinions upon this point, and of enlightening our benighted readers, that we pen this article. Some side issues have come up in this matter. Dr. Cartwright has drawn most of his physiological arguments from Holy Writ. Moses and the prophets it seems were in advance of their times. We were aware that the sanitary system of Moses for the government of the camp of Israel, was a very perfect one; and Dr. Cartwright now informs us that "he (Moses) was the greatest physiologist of any age." Now some of our western editorial brethren have been imprudent enough to declare, that with all due deference to Moses, they prefer Dr. Carpenter. Whereupon some other medical editors sound the alarm. The authenticity of scripture is attacked, and the "battle of the evidences" is to be fought in the medical journals.

Now here is a very pretty quarrel. One would guess there was a "lady in the case" somewhere. Dr. Cartwright comes up vigorously in defense of the new theory. Gallantry, religion, and scientific zeal alike urge him on. From our boyhood we have felt an irresistible impulse to have a share in any "muss" that is going on, and though we do not suppose that all this matter is going to save a patient for any man, or do any other practical good, yet the thing has such an admirably scientific tone, that we like it. We appeal to an intelligent profession, if there is in all Dunglison, a longer word than "hæmatokinetic." Great names are cited as on the point of conversion. Dr. Bennet Dowler is represented as struggling against conviction in the most affecting manner; and all the good names in New Orleans are introduced as in a somewhat similar condition of stubborn unbelief—averting their eyes from the truth, and refusing to follow the lead of a woman, because, alas, she *is* a woman!

We have not seen anything from Mrs. Willard's pen having any special bearing on the theory itself—her own articles in the journals being confined to sundry explanations as to her feelings of thankfulness at having been made the feeble instrument by which the medical world was to be shaken; and her dread of the persecution which she, in common with Luther, Galileo, and Jenner, was to endure.

Dr. Cartwright is the mouthpiece, and to his arguments we will first direct our attention. In the first experiment cited by Dr. C., a "Nilotic saurian," a "*Niliaca fera,*" *alias* an alligator, was made what the Dr. calls "the messenger of the mandate for your (Mrs. Willard's) enrollment among the immortals." The experiment was briefly this:—An alligator was killed by tying the trachea; the walls of the chest were then removed, artificial inflation commenced, "and soon a faint quivering of moving blood in the diaphanous veins of the lungs began to be seen, * * * * and at length the blood began to run in a stream from the lungs into the quiescent heart. Then the heart began to quiver, and soon to pulsate." Finally, the animal vindicated his vitality by "vigorous exertions to get loose, biting and snapping at everything." Years ago we performed this same experiment on an opossum, with equal success. Dr. C. draws from it the inference, that "the *primum mobile* of the circulation, and chief motive powers of the blood are in the lungs, and not in the heart." Dr. C. does not resort to the theory of molecular motion to account for this. The evolution of caloric he considers a sufficient cause; the blood moving in the lungs like water in a tea-kettle It has long been our conviction, that in every misconception or error, there is a germ of truth. It is not contended that the heart acts independently of the other vital processes in its propulsive functions. No one has ever supposed the heart to be capable of carrying on the circulation for any length of time, without the aid of the lungs. But it is an error to suppose, that because in a state of asphyxia the lungs are first to show signs of motion, that they are really the *primum mobile.* The heart acts during fœtal life while the lungs are quiescent—*it* inaugurates the movement, and blood must be sent from the heart to the lungs, before the necessary chemical changes can occur. We will take the other case, that of asphyxia. Here blood is present in the lung—it meets with oxygen—a motion occurs; it is slowly propelled to the quiescent heart. Does this prove the motive power in the lungs, or in the blood itself? Molecular motion, the *effect of pressure* upon the capillary vessels of the lungs by the inflation of the air cells, seems as likely as any other cause to induce this motion. The healthy performance of the circulatory function depends on many conditions. Different densities, occasioning different pressures, seem to lie at the bottom of the matter. The movement of muscles furnishes this condition; atmospheric pressure within the lungs, against the antagonistic action of the thoracic walls, also supplies it. Again, this condition of different densities, blocks Mrs. Willard's theory at the heart. Suppose that by caloric the first impulse is given in the parenchyma of the lung, the blood reaches the heart, and encounters these muscular motions which expels it in a direction contrary to that communicated by the lung. This "chief motive force" then extends no farther than the pulmonary veins. Reverse the experiment; put a stop to heart motion, and then restore it. The lungs stop with the heart. By irritation of the heart, by galvanism, (or as we have always seen it done, by simply pinching its substance,) you restore its action and the blood is propelled from the heart to the lungs. Here you are on the other horn of the dilemma—the heart is, in this case as in fœtal life, the *first* moving force; it requires no argument to prove it the *chief* power. One experiment is as good as the other, and both involve the same uncertainties.

After a careful perusal of Dr. Cartwright's subsequent articles and experiments, we are unable to find any new arguments in support of this theory. He claims that all resuscitations of drowned people, and still-born infants, are vindications and proofs of its truths. He might have added, that every fœtal heart pulsating, *in utero*, is a contradiction of it.

He then quotes Lord Bacon, and Newton, to the effect that discoveries are to be judged by their results. With marvelous complacency he next assumes, that all ventilation, all dumb-bells, and gymnastic exercises; all those health-giving processes which act by giving vigor to the respiratory functions, are the legitimate derivatives of Mrs. Willard's theory — that they are its fruits.

So much for the scientific features of this hypothesis. The side issues to which we have alluded are also worthy of notice. In what we have said of "Moses and the prophets," no irreverence was intended, and no unbelief indicated. But we always (for the sake of that religion which has given us so many blessings) deprecate and protest against the lugging in of scriptural texts, as arguments in favor of a scientific theory. Thus Dr. Cartwright asserts that Mrs. Willard's is "the physiological doctrine taught in the Pentateuch: 'The life of the flesh is in the blood.'"—(Lev. xvii. 11.)

We have elsewhere quoted an assertion of the great physiological acquirements of Moses. This is simply absurd. Truth, if it is truth, should sustain itself. The Bible was intended as a rule of faith and practice, and not as a compendium of scientific truth. An editor of a western medical periodical, declares himself ready to buckle on the armor, and fight the battle of the evidences in the pages of his journal. When we have settled some mooted points in pathology and therapeutics; and when we have physiological knowledge enough to know positively whether science contradicts inspiration, we shall be ready to go into a theological discussion. Thus far, as seeming contradictions to Bible truth have arisen in scientific research, farther investigation has reconciled them. We trust this will always be the case. In the mean time, we shall read our Bible for the benefit of our heart, rather than our head, and seek elsewhere for scientific truth. S. B. H.

